# Acute Effects of High- Versus Low-Elevation-Gain Trail Running Training on Static and Dynamic Postural Balance in Young Trail Runners

**DOI:** 10.3390/jfmk11040394

**Published:** 2026-10-01

**Authors:** Carlos Castellar, Abraham Blánquez, Alejandro García-Giménez, Miguel Lecina

**Affiliations:** 1 ENFYRED Research Group, Faculty of Health and Sports Sciences, University of Zaragoza, 22002 Huesca, Spain; castella@unizar.es; 2 Faculty of Health and Sports Sciences, University of Zaragoza, 22002 Huesca, Spain; ablanquez23@gmail.com; 3 Faculty of Health, Universidad de San Jorge, Autovía A-23, km. 299, 50830 Villanueva de Gállego, Spain; agarciagimenez@usj.es

**Keywords:** adolescence, exercise interventions, endurance training, injury prevention, muscle alteration, postural balance, physical activity, sports science

## Abstract

**Background:** Trail running is a sport characterized by taking place in natural environments and requiring constant adaptation to irregular terrain, making postural balance crucial for preventing falls and coping with the coordinative demands of these races. **Objectives:** This study aimed to analyze the acute effects of elevation on dynamic and static balance in young trained trail runners. **Methods:** Fifteen highly trained young athletes (eight females, seven males; age 16.8 ± 2.14 years) completed two similar training sessions except for elevation gain (1000 m vs. 15 m, flat). Static balance was evaluated using the Balance Error Scoring System (BESS), and dynamic balance was assessed with the Y Balance Test (YBT) before (pre) and after each session (post) using 2 × 2 (Time × Terrain) repeated-measures ANOVA. **Results:** BESS errors increased in both sessions but more after elevation (+3.33 vs. +1.20 errors; interaction *p* < 0.001; post-session 15.67 ± 1.23 vs. 12.67 ± 1.23). The YBT composite index showed no significant differences between elevation and flat sessions (83.32 ± 7.92 vs. 83.79 ± 7.59, *p* = 0.763) or in the interaction (*p* = 0.057). **Conclusions:** These findings highlight the need for specific strategies to preserve postural balance—especially static balance—during training and after trail races and support taking elevation gain into account when programming training load in young trail runners.

## 1. Introduction

Trail running (TR) has emerged in recent years as a discipline of increasing scientific interest within both competitive and recreational domains [1]. These races take place in a natural environment characterized by uneven terrain and significant accumulated elevation gain (both positive and negative), which imposes pronounced physiological and neuromuscular demands on runners’ bodies [2]. The continual alternation between steep ascents and descents forces the body to adapt both at the physiological and neuromuscular levels [3]. While ascents demand high aerobic capacity and efficient recruitment of the extensor muscles, descents imply a mastery of eccentric control, as well as effective proprioceptive processing to respond to unpredictable stimuli from the terrain [4,5]. The biomechanical demands of these kinds of races affect the postural balance (PB) which becomes a critical skill, not only for optimizing movement efficiency but also for reducing the risk of falls and injuries [6].

PB is defined as the ability to maintain the center of mass within the base of support, either during stability (i.e., static balance) or mobility (i.e., dynamic balance) [7]. In sport, PB contributes to greater technical efficiency, lower energy expenditure and a lower risk of injury [8] and in the long term is vital to acquiring strength [9] and preventing inter-limb asymmetry of the athletes [10]. Trail runners, especially on technical descents, must constantly activate their postural control mechanisms to adjust their center of gravity, react to uneven terrain and avoid falls; loss of balance can compromise both performance and safety [11]. Two types of balance are distinguished: static, which refers to the ability to keep the body in a stable position without displacement, and dynamic, which involves maintaining postural control during movement [12].

In TR, PB imbalance is mainly caused by neuromuscular fatigue [13] and, particularly when induced by eccentric and prolonged exertion, has been shown to negatively impact balance, compromising both the static and dynamic stability [14,15]. Neuromuscular fatigue is defined as the reduction in the ability of a muscle or muscle group to generate force, either due to central (nervous system) or peripheral (muscle) causes [16]. In the context of TR, this fatigue is intensified by the prolonged duration of the effort and by the complexity of the terrain [17]. Several studies have shown that both general and localized fatigue negatively affect postural balance [18,19]. In particular, fatigue induced by eccentric contractions, such as those that occur on descents, is associated with a greater loss of stability [20,21]. It has also been observed that the effects of fatigue on dynamic balance can be more significant, compromising the athlete’s ability to respond effectively to stimuli from the environment [22]. This fatigue can manifest itself locally—affecting specific muscle groups—or generally, interfering with the central mechanisms of motor control and sensory perception [18].

Among the most widely used tools for balance assessment in research and clinical practice are the Balance Error Scoring System (BESS) and the Y Balance Test (YBT). The BESS is a validated test to evaluate static balance through different postures on stable and unstable surfaces [23]. The YBT can measure dynamic balance through the reach of the foot in three directions and has been widely used in young sports populations [24]. These instruments have been used in research on postural control under fatigue conditions, proving to be sensitive to changes in stability after demanding physical exertion [25].

Despite this growing body of evidence on the effects of fatigue on postural control, gaps remain regarding how trail-running-specific topographic features—particularly cumulative elevation gain, defined as the sum of meters ascended and descended—influence postural performance. This gap is especially relevant for young populations still undergoing neuromotor development [26]. Postural control does not reach full maturity until adolescence, developing at an individualized pace closely tied to biological maturation and proximity to peak height velocity [27]; furthermore, sport specialization during this period has been associated with altered lower-limb biomechanics and neuromuscular control across the pubertal transition [28]. This ongoing postural maturation suggests that young athletes may respond differently from adults to the acute neuromuscular demands imposed by variable and technical terrain, yet this possibility remains largely unexplored [29]. To date, only a small number of studies have addressed this question directly. Drum et al. (2023) compared TR with asphalt running and found that trail runners exhibited more efficient neuromuscular adaptations for maintaining postural control, which the authors attributed to repeated exposure to varied mechanical stimuli from the terrain [30]. While this finding points to terrain-specific adaptive potential, it was not examined in youth populations, and no study to date has specifically assessed how cumulative elevation gain acutely affects postural balance in adolescent or technification-stage trail runners. Almutairi et al. (2025) found that young athletes exhibit neuromuscular adaptations distinct from those of adults, potentially related to ongoing growth and development processes affecting PB; however, this was examined only in road running contexts [26]. Likewise, previous research in more general contexts has shown a decrease in PB following fatigue exercise [31], which allows us to infer that mountainous environments, which are more demanding due to continuous changes between downhill and uphill, could aggravate this loss of balance in adolescent runners as it occurs in their counterparts [32].

To address this gap, the present study aims to analyze and compare the acute effects of training sessions with high versus low accumulated elevation gain on postural balance—both static and dynamic—in young runners from the “Aragoneses trail running technification group.” Through an experimental pre- and post-effort evaluation design, we hypothesize that elevation gain produces greater alteration in balance dynamic and static in young ultra trail runners. This work seeks to generate evidence applicable to the design of safer and more effective training protocols that account for the real demands of the natural environment in which this discipline takes place.

## 2. Materials and Methods

### 2.1. Study Design

The design of the present study is a quasi-experimental, intra-participant, pretest-post-test type, in which each participant is evaluated before and after two training conditions differentiated by elevation gain. The categorization of these conditions was carried out by means of an ad hoc division, taking as a reference the average accumulated slope and distances of the competitions in which the runners in the sample usually participate. To account for differences in distance and elevation gain between conditions, the km-effort was calculated as the sum of the horizontal distance (km) and the positive elevation gain (m) divided by 100, following the methodology adopted by the International Trail Running Association (ITRA) [33].

This methodology allows us to compare the effects of the type of terrain on PB within the same individual, reducing interindividual variability and increasing the statistical sensitivity of the analysis. This Final Degree Project has been evaluated and approved by the Research Ethics Committee of the Autonomous Community of Aragon (CEICA), which issued a favorable opinion at its meeting on 4 June 2025 (Minutes Nº11/2025), under code PI25/144. The proposal presented, entitled “Comparative analysis of the unevenness in balance in mountain races in young runners”, was considered in accordance with ethical principles and current legislation, guaranteeing the confidentiality of the data, the correct obtaining of informed consent and the appropriate use of the necessary resources. In addition, all participants, or their legal guardians in the case of minors, signed the informed consent. The data were pseudonymized and managed confidentially, in compliance with the current regulations on the protection of personal data of the University of Zaragoza.

### 2.2. Sample

The sample was composed of 15 young runners (8 boys and 7 girls), belonging to the “Aragones mountain running technification group”, age 16.80 ± 2.14. All participants had at least 2 years’ previous experience in TR (4.07 ± 1.67) and were in good health, with no active musculoskeletal injuries or balance disturbances in the past six months. The full data of the participants is presented in Table 1.

### 2.3. Study Criteria

#### 2.3.1. Inclusion

Inclusion criteria were as follows: (a) males and females aged between 14 and 20 years old; (b) no injuries or neurology alterations; (c) members of the technification group of the Aragones mountaineering federation; (d) absence of recent injuries (<6 months) or limiting neurological alterations; (e) participants who had signed the informed consent or whose legal guardian had done so (in the case of minors).

#### 2.3.2. Exclusion

Exclusion criteria were as follows: (a) presence of injury or disease affecting the musculoskeletal or neurological system and (b) failure to comply with scheduled sessions.

### 2.4. Procedures

The study was carried out during the official training camps of the technification group in two locations: the training with the highest slope in Agüarón (Zaragoza) and the training with the lowest slope in a flat area (Galachos de Juslibol, Zaragoza) seven days later. All participants received the same training instructions 48 h prior to the measurement sessions so that they arrived in optimal recovery conditions. Each participant carried out two different sessions:

- Session A: training with high elevation gain (1000 m positive/negative) and 12 km. completion time was 120 ± 26′54″ temperature 6 °C and 65% humidity.
- Session B: training with low elevation gain (15 m positive/negative) and 18 km. Completion time was 90’± 11′05″, temperature 3 °C and 80% humidity.

Both sessions were monitored under technical supervision, and the kinematic and physiological parameters were recorded with a Garmin^®^ Fenix 6 smartwatch and were carried out in the afternoon.

#### 2.4.1. Phases of the Protocol

Pre-training evaluation: The BESS and YBT tests were performed under controlled conditions and without prior fatigue just before starting the training session.

Training session: The group went out in the mountains according to the slope planned for the session.

Post-training evaluation: Immediately after the end of the training, the balance tests were repeated, and the perceived fatigue was recorded.

**Figure 1:**
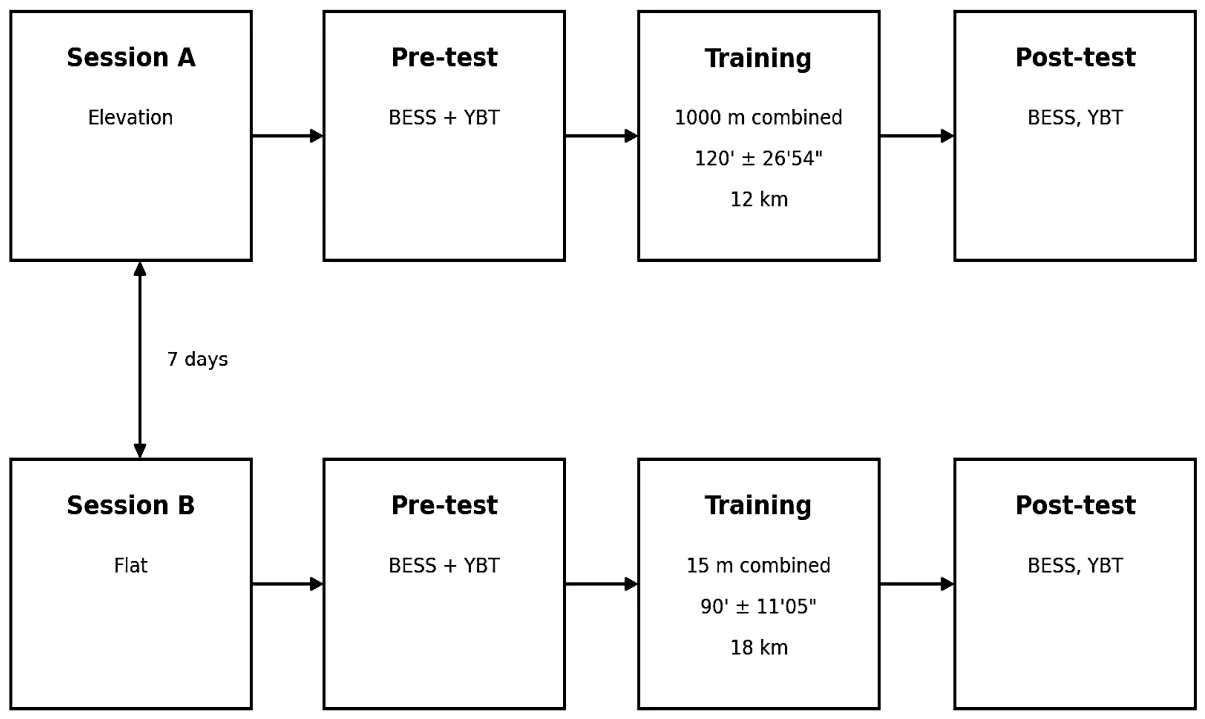
Protocol of the study.

The order of the sessions was crossed to balance the possible effect of the order, and the two training sessions were separated by 7 days to ensure a full recovery. Figure 1 details the full protocol.

#### 2.4.2. Dynamic Balance Assessment

The YBT was used in this study as a tool to assess the dynamic balance of the participants, following the standardized protocol described by Plisky et al. (2006) [34]. This test, widely validated in the sports field, allows the detection of functional asymmetries between lower limbs through the analysis of the reach of the contralateral limb in different directions, maintaining balance on one leg.

**Figure 2:**
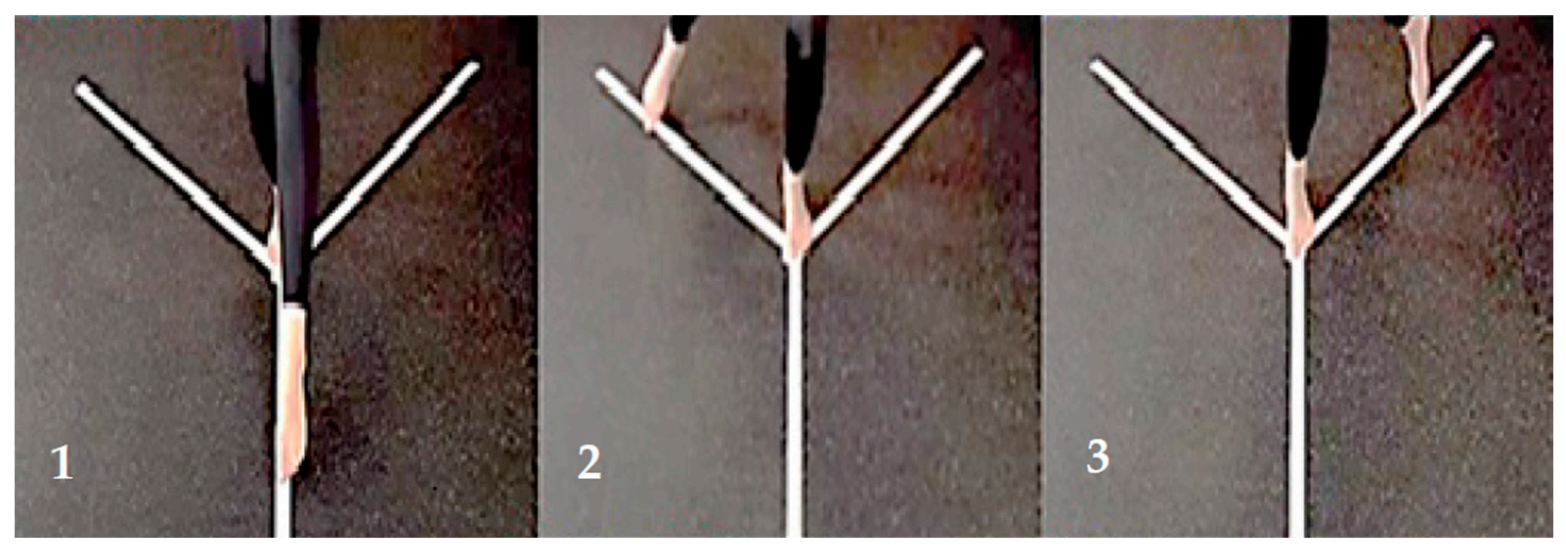
Y Balance Test directions. (1) YBT PA, (2) YBT PM, (3) YBT PL.

The test was carried out in an enclosed space, with a flat and non-slip floor free of obstacles. To carry it out, a system of tape measures adhered to the ground was used, forming a “Y” shape, with three directions of reach: anterior (YBT PA), posteromedial (YBT PM) and (YBT PL) posterolateral, separated from each other by angles of 135°. This arrangement was designed to ensure the repeatability of the test and the accuracy in the measurement of the distances reached [35], as shown in Figure 2.

Prior to execution, the length of each leg was measured from the anterior superior iliac spine to the medial malleolus, following the methodology established in the literature [35]. This measurement was later used to normalize the distances reached, thus allowing an objective comparison between participants with different height and length of the lower limbs.

Each participant performed the test barefoot, with their hands resting on their hips and their supporting foot placed at the central vertex of the Y in the floor (see Figure 2). From this position, the participant was asked to reach, with the free foot, the greatest possible distance in each of the three directions, without losing the balance or resting the foot on the ground during the movement (Figure 2). Each attempt was recorded individually, and those in which there was loss of balance, premature contact of the foot with the ground or separation of the hands from the hips were considered invalid.

Three valid attempts were made per direction and leg, selecting the greatest distance reached for analysis. Subsequently, the values were normalized using the following formulas:

- (Range 1 + Range 2 + Range 3)/3 = average distance in each direction measured in centimeters.
- (Average distance in each direction ÷ leg length) × 100 = relative normalized distance in each direction (%)
- (Normalized YBT PA + normalized YBT PM + normalized YBT PL)/3 = range composite distance (%)

In addition, a composite index (CI) was calculated for each leg, integrating the three test vectors according to the following equation:

CI (%) = (YBT PA + YBT PM + YBT PL)/(3 × Leg Length) × 100

The values obtained were analyzed at the intra-individual level (pre-post) and between conditions (high and low slope), considering as possible risk indicators a difference of more than 4 cm between lower limbs in the previous direction or composite indices below 94%, as established in previous studies [36].

#### 2.4.3. Static Balance

**Figure 3:**
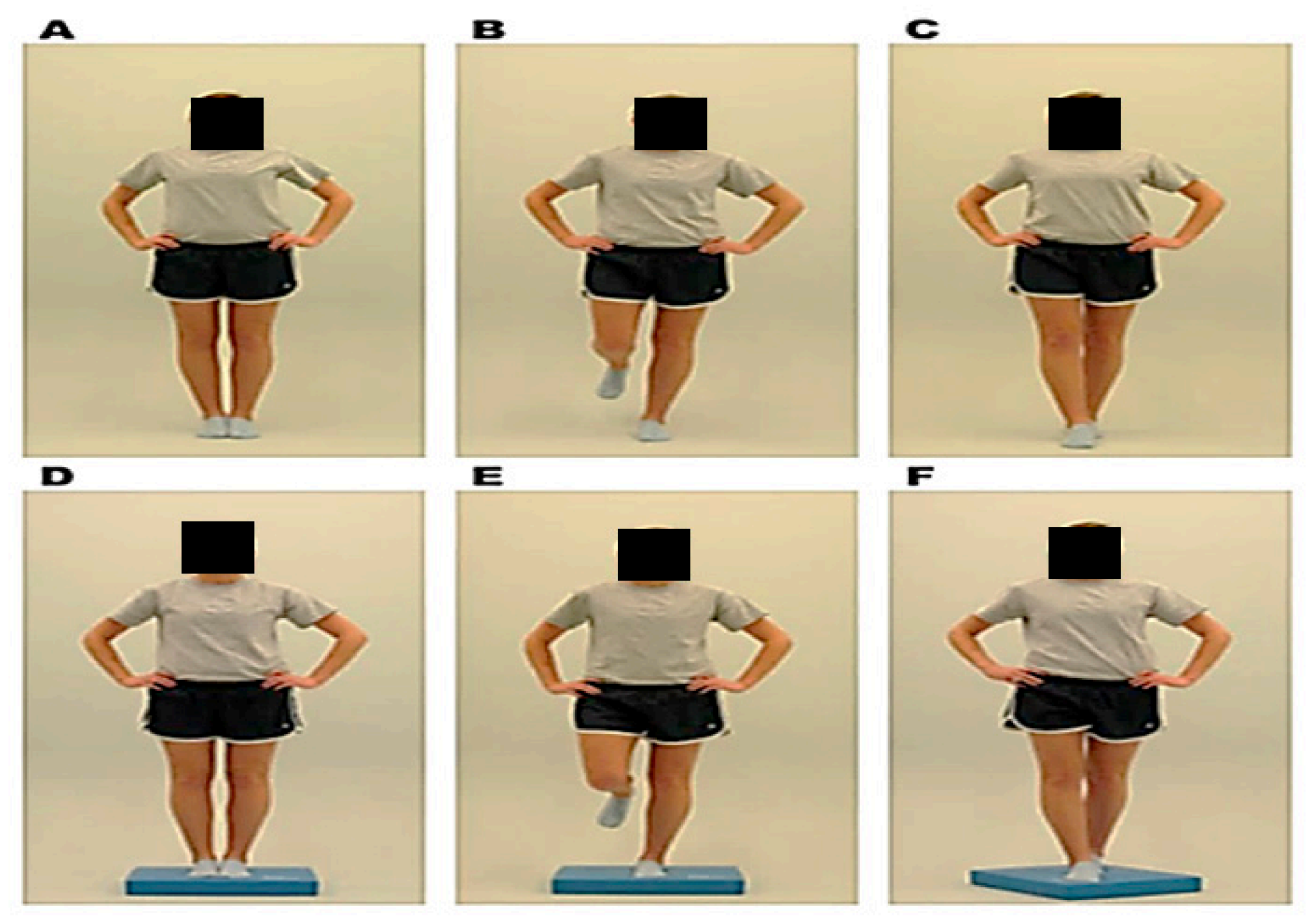
Graphic description of the six positions for BESS measures.

The Balance Error Scoring System (BESS) is a widely used test to evaluate static postural balance, especially in sports and clinical settings. It is characterized by being a simple, economical and reliable tool for detecting alterations in postural control that may arise from fatigue, injuries or mild neuromuscular dysfunctions [37,38]. The BESS protocol includes six different conditions that combine three body postures—standing with feet together, semi-tandem (one foot slightly forward of the other), and tandem (one foot directly in front of the other, heel with toe)—on two types of surfaces: a firm one (such as the ground) and an unstable one (usually a foam pad) (Figure 3). In this condition, the participant should hold the posture for 20 s, with the eyes closed and the hands placed on the hips.

During that time, the assessor records postural errors, which reflect losses of balance control. Each mistake counts as one point in the scoring system. An error is counted each time the participant performs the following actions (Figure 3):

- Raising their hands from their waist.
- Opening their eyes.
- Taking a step, jumping or falling out of the pose.
- Lifting their forefoot or heel off the ground.
- Flexing the hip excessively (>30°) or trunk.
- Staying out of position for more than 5 s.

A maximum of 10 errors can be accumulated in each of the six conditions. If the participant loses their balance to the point of not being able to hold the posture for more than 5 s, the maximum score of 10 errors is automatically assigned for that condition, and that part of the test is completed. The total score of the BESS is the sum of the errors made in all conditions: the greater the number of errors, the worse the postural control. This test has been used in numerous studies to assess how exercise-induced fatigue affects balance.

### 2.5. Statistical Analysis

Data were analyzed using the program SPSS 26.0 (Statistical Package for The Social Sciences Inc., Chicago, IL, USA) with a significance value set at 5%. Two dependent-samples repeated-measures designs were used to compare dynamic balance (Youth Balance Test, YBT) and static balance (Balance Error Scoring System, BESS) between the flat and elevation training conditions and time condition (pre and post), with each of the 15 participants serving as their own control. Because each factor has only two levels, the sphericity assumption does not apply, and each effect was tested as a one-sample *t*-test on its contrast score (*F* = *t*^2^, df = 1, 14): Time (mean of Post conditions minus mean of Pre conditions), Terrain (elevation minus flat), and interaction (Pre-to-Post change in elevation minus Pre-to-Post change in flat). Effect sizes are reported as partial eta squared (*η_p_*^2^), with values of 0.01, 0.06, and 0.14 interpreted as small, medium, and large, respectively.

## 3. Results

### 3.1. Dynamic Balance (YBT)

Descriptive statistics and ANOVA results for the YBT composite score are shown in Table 2. No significant main effect of Time, *F*(1, 14) = 0.35, *p* = 0.564, *η_p_*^2^ = 0.02, or Terrain, *F*(1, 14) = 0.09, *p* = 0.763, *η_p_*^2^ = 0.01, was found. The Time × Terrain interaction showed a large effect size but did not reach statistical significance, *F*(1, 14) = 4.31, *p* = 0.057, *η_p_*^2^ = 0.24. Table 2. 

Simple-effects analyses showed that the composite score changed by +2.66 points on elevation (95% CI [−2.37, 7.69], *t*(14) = 1.14, *p* = 0.275, *d_z_* = 0.29) and by −0.62 points on flat ground (95% CI [−3.44, 2.20], *t*(14) = −0.47, *p* = 0.644, *d_z_* = −0.12). Between terrains, the composite score on elevation was 2.12 points lower than on flat ground at Pre (*t*(14) = −1.11, *p* = 0.287, *d_z_* = −0.29) and 1.16 points higher at Post (*t*(14) = 0.74, *p* = 0.471, *d_z_* = 0.19). After Bonferroni correction, all padj values were 1.00. The Shapiro–Wilk test did not indicate significant deviations from normality (*p* = 0.066–0.984), and the Wilcoxon signed-rank test yielded consistent results (Time, *p* = 0.762; Terrain, *p* = 0.890; interaction, *p* = 0.083).

### 3.2. Static Balance (BESS)

The descriptive statistics and ANOVA results are shown in Table 3. The ANOVA revealed a significant main effect of Time, *F*(1, 14) = 58.53, *p* < 0.001, *η_p_*^2^ = 0.81, with the number of errors increasing from Pre (11.90) to Post (14.17), and a significant main effect of Terrain, *F*(1, 14) = 122.01, *p* < 0.001, *η_p_*^2^ = 0.90, with more errors on elevation (14.00) than on flat ground (12.07). The Time × Terrain interaction was also significant, *F*(1, 14) = 37.14, *p* < 0.001, *η_p_*^2^ = 0.73, indicating that the Pre-to-Post increase in errors was 2.13 errors larger on elevation than on flat ground. Simple-effects analyses showed that errors increased from Pre to Post on elevation (+3.33 errors, 95% CI [2.50, 4.16], *t*(14) = 8.63, *p*_adj_ < 0.001, *d*_z_ = 2.23) and, to a lesser extent, on flat ground (+1.20 errors, 95% CI [0.57, 1.83], *t*(14) = 4.05, *p*_adj_ = 0.005, *d*_z_ = 1.05).

## 4. Discussion

The results of the present study reinforce the initial hypothesis that elevation gain negatively affects postural balance in young trail runners as has been shown in adolescents in other sports [28] and in the general population in TR [39]. To our knowledge this study is the first to analyze acute PB effects in TR and in adolescents.

The impairment found was more pronounced in static balance, assessed by the BESS protocol, where an average increase of three errors was observed after elevation training compared with flat training (15.53 ± 1.23 vs. 12.67 ± 1.33, *p* < 0.001). This difference reflects a clear deterioration in immediate postural control, attributable to fatigue created after completing a training session with elevation in uneven terrain. In the case of dynamic balance, YBT values remained virtually unchanged after sessions with higher topographic load. Consistent with this, no statistically significant differences were observed between flat and elevation conditions when comparing pre- vs. post-session CI values (83.79 ± 7.59 vs. 83.32 ± 7.92, *p* = 0.763). Accordingly, none of the three components of CI, YBT PA, YBT PM and YBT PL showed statistically significant differences either. These results may be explained by the short duration of the training sessions in both flat and elevation conditions.

This pattern coincides with the results found by D’Emanuele et al. (2021) in a systematic review which included 15 studies in running and did not find significant alterations in static balance in athletes and only in recreational runners found some evidence of this decrease in static balance (five of the seven studies included) [9]. Similarly, Almutairi et al. (2025) in a randomized trial with young athletes in a treadmill running found that static balance worsened but dynamic balance measured through the YBT did not show statistical differences [26]. Similarly, Khassetarash et al. (2022) used downhill running as a model to analyze neuromuscular, biomechanical and energetic adjustments, showing that repeated bouts of downhill running induce specific neuromuscular responses and modifications in running biomechanics [40]. Recent studies like De Maio et al. (2026), have found that the YBT may not fully capture changes in the motor strategies used to maintain dynamic postural stability, like in individuals with ankle instability; however, the population of this study were healthy [41]. These findings may support the interpretation that the mechanical load generated by descents is not only metabolically demanding but also produces alterations in muscle function and movement control that ultimately affect PB.

Taken together, these results confirm a broader pattern: previous studies have shown that both localized and general fatigue negatively affect dynamic postural control. Johnston et al. (2018) reported that maximal anaerobic fatigue reduced normalized YBT scores in the anterior, posteromedial and posterolateral directions [42]. Although their protocol was not specific to TR, their results support the sensitivity of the YBT for detecting acute impairments in dynamic balance after intense physical exertion.

The BESS results reflect the static balance, and the results found in our study were similar to other studies which have analyzed static balance in running. Wilkins et al. (2004) measured BESS in a randomized trial, finding more errors in the intervention group than in the control (16.93 ± 4.32 vs. 14.36 ± 4.73), but this study included jumps and not running [43]. Exploring the nature of the movement, Munkasy & Buckley (2025) in a comparative study analyzing the Bess parameter in running flat and in a drop jump; in 52 active participants, there were greater decreases in drop jump group but no statistical differences between both groups [44]. Despite the reliability of the BESS score the heterogeneity of the different sports and population included prevented researchers from identifying the causes of the variety of the results found. Despite this disparity the point in common from all the studies which assessed PB is that all concluded that the fatigue is the main culprit in the alterations found in static and postural balance [13,43,45,46].

Previous studies have shown that both localized and general fatigue negatively affect postural control [18,47], especially in environments with high mechanical variability such as TR [48]. Our findings reaffirm this line of evidence: while static balance showed a more uniform response to eccentric effort, dynamic balance was more conditioned by individual factors such as training history, level of neuromuscular maturation, and functional symmetry [13,23,49].

From a practical point of view, the usefulness of the BESS test is confirmed as a reliable tool for identifying acute postural alterations after intense running sessions, in agreement with previous studies [26,45]. On the other hand, the YBT allows the detection of small functional deficits that could go unnoticed in static conditions, which makes it a key tool for specific training planning [24].

### Limitations

Several limitations should be acknowledged when interpreting the findings of the present study. First, the sample size was small (*n* = 15), and the exposure period was limited to two sessions, which may limit the generalizability of the findings. Although the BESS and the YBT tests are reliable and widely used for assessing postural balance, their applicability across different sports and exercise contexts may be influenced by the specific demands of each activity.

Furthermore, trail running races are characterized by considerable heterogeneity in terms of duration, elevation gain, number of stages, terrain characteristics, and environmental conditions. This variability makes the classification and standardization of trail running events challenging and may contribute to the heterogeneity observed in the scientific literature regarding their effects on runners’ physiological and neuromuscular responses. In addition, the inclusion of more comprehensive measures of training load would have strengthened the methodological assessment of the study and facilitated a more accurate interpretation of the observed responses.

Given the increasing popularity of trail running and its potential effects on young runners, further research is warranted, particularly in adolescent and young populations. Future studies should include larger samples, longer exposure periods, and a more comprehensive assessment of training load and race characteristics. Longitudinal designs may also help to clarify the effects of repeated exposure to trail running on postural balance and other neuromuscular outcomes in young runners.

Despite these limitations, to the best of our knowledge, this is the first study to investigate postural balance in adolescent trail runners following trail running exposure. Therefore, the present findings provide preliminary evidence regarding postural balance responses in this population and may serve as a basis for further research in this emerging field.

## 5. Conclusions

The present work has shown that the elevation gain accumulated during training significantly influences the postural balance of young trail runners, especially in static balance. Through a rigorous methodology based on a within-subject (crossover) design and the use of validated assessment tools—the BESS for static balance and the YBT for dynamic balance—it has been shown that exposure to prolonged and technically demanding efforts generates an acute decrease in postural stability.

An increase in errors made in the BESS test was observed after training with high elevation gain, suggesting a loss of postural control derived from the continuous changing between concentric and eccentric muscle contractions which increase fatigue. This effect was particularly marked under static conditions, indicating a clear impairment of the neuromuscular system’s ability to maintain stability after downward loading stimuli.

In relation to dynamic balance, the YBT results showed greater variability between participants after the uneven session. Although the mean differences did not reach statistical significance, this difference suggests that the impact of fatigue on dynamic control is more dependent on individual factors, such as the degree of neuromuscular maturation, functional symmetry and previous experience in the technical field.

From an applied perspective, these findings highlight the need to incorporate the specific work of balance as a structural component in the physical preparation of young mountain runners. This training should include both static and dynamic tasks specially designed to be implemented in states of fatigue, with the aim of simulating real running conditions and strengthening the compensatory mechanisms of postural control.

In conclusion, postural balance should be considered a critical ability in the development of TR runners, especially in the face of loads with a high elevation component. TR adolescent runners’ training, continuous evaluation and integration into real fatigue contexts are key elements for effective and safe technification in the long term considering the growth and maturational characteristics of adolescence.

## Figures and Tables

**Table 1:** Characteristics of the study group.

Participants	Sex	Height	Weight	Age	TR Exp.
	(f, m)	(cm)	(kg)	(y)	(y)
Participant 1	f	164	54.2	16	3
Participant 2	m	190	72.8	20	6
Participant 3	m	178	63.5	18	5
Participant 4	m	174	61	17	4
Participant 5	f	169	56.8	16	3
Participant 6	m	195	76.5	20	7
Participant 7	f	165	55.1	15	3
Participant 8	m	176	62.4	18	5
Participant 9	m	187	70.2	19	6
Participant 10	m	183	67.4	19	6
Participant 11	f	154	46.8	14	2
Participant 12	f	153	45.9	14	2
Participant 13	f	145	40.6	14	2
Participant 14	f	170	57.6	17	4
Participant 15	f	159	50.2	15	3
AV		170.8	58.73	16.80	4.07
SD		14.50	10.38	2.14	1.67

AV = average; cm, = centimeters; kg = kilograms; m = male; f = females: SD = standard deviation; y = years; TR exp = experience in trail running races; y = years.

**Table 2:** Dynamic balance measured with the YBT as a function of Time and Terrain (*n* = 15).

Terrain	Pre, *M* (*SD*)	Post, *M* (*SD*)	Δ (Post − Pre) [95% CI]
Elevation	81.98 (9.44)	84.65 (8.80)	+2.66 [−2.37, 7.69]
Flat	84.10 (7.86)	83.49 (8.15)	−0.62 [−3.44, 2.20]
Effect	*F*(1, 14)	*p*	*η_p_* ^2^
Time	0.35	0.564	0.02
Terrain	0.09	0.763	0.01
Time × Terrain	4.31	0.057	0.24

*M* = mean; *SD* = standard deviation; CI = confidence interval; *η_p_*^2^ = partial eta squared. Composite score = (ANT + PM + PL)/(3 × limb length) × 100, averaged across both limbs. Higher values indicate better dynamic balance. Time × Terrain denotes the interaction effect.

**Table 3:** Static balance (BESS, number of errors) as a function of Time and Terrain (*n* = 15).

Terrain	Pre, *M* (*SD*)	Post, *M* (*SD*)	Δ (Post − Pre) [95% CI]
Elevation	12.33 (1.45)	15.67 (1.23)	+3.33 [2.50, 4.16]
Flat	11.47 (1.46)	12.67 (1.23)	+1.20 [0.57, 1.83]
Effect	*F* (1, 14)	*p*	*η_p_* ^2^
Time	58.53	<0.001	0.81
Terrain	122.01	<0.001	0.90
Time × Terrain	37.14	<0.001	0.73

*M* = mean; *SD* = standard deviation; CI = confidence interval; *η_p_*^2^ = partial eta squared. Higher BESS scores indicate poorer balance. Time × Terrain denotes the interaction effect.

## Data Availability

Further inquiries can be directed to the corresponding author.

## Abbreviations

The following abbreviations are used in this manuscript:

TR	Trail Running
YBT	Y Balance Test
BESS	Balance Error Score System
YBT PA	Y Balance Test Postanterior
YBT PL	Y Balance Test Postlaterior
YBT PM	Y Balance Test Postmedial
